# Role of Combined Use of Adiponectin and hsCRP in Cardiovascular Risk in Pediatric Neurogenic Bladder

**DOI:** 10.3390/children12060748

**Published:** 2025-06-09

**Authors:** Joanna Bagińska-Chyży, Alicja Szymańska, Agata Korzeniecka-Kozerska

**Affiliations:** Department of Pediatrics and Nephrology, Medical University of Białystok, 17 Waszyngton Str., 15-274 Białystok, Poland; iklinped@umb.edu.pl (A.S.); agatakozerska@poczta.onet.pl (A.K.-K.)

**Keywords:** neurogenic bladder, adiponectin, high-sensitivity CRP, cardiovascular risk

## Abstract

**Background/Objectives:** Myelomeningocele (MMC) is the most severe form of spina bifida, often accompanied by impaired motor function due to paralysis of the lower limbs, as well as neurogenic bladder (NB). These factors may contribute to nutritional disorders and cardiovascular diseases (CVDs) in the future. High-sensitivity CRP (hsCRP) is a positive marker of unstable atherosclerotic plaques and is commonly used in the diagnosis of CVDs. Adiponectin has an opposite, anti-inflammatory function. The aim of this study was to assess the risk of CVDs in a group of children with NB and a control group, based on serum levels of adiponectin, hsCRP, and lipid profiles. **Methods**: A prospective clinical estimation based on 87 children (67 NB, 20 control group) was conducted. Data collected from medical histories included the following: sex, age, anthropometric parameters (height, weight, BMI), level of spinal lesion, and activity according to Hoffer’s scale. Lipid profile values (cholesterol, HDL, LDL, triglycerides) were assessed using standard blood sample tests. hsCRP and adiponectin were measured using an ELISA kit. **Results**: A comparison of adiponectin and hsCRP levels revealed statistically significant differences between the NB group and the control. Additionally, significant correlations were identified between BMI and the biomarkers: hsCRP was positively associated with BMI, whereas adiponectin exhibited a negative association. The highest concentrations of hsCRP were detected in MMC patients with a Th lesion level and in non-walker patients. **Conclusions**: Elevated hsCRP may reflect increased cardiovascular risk in children with NB. While adiponectin levels were also altered, their association with cardiovascular risk appears more complex and may involve additional metabolic mechanisms.

## 1. Introduction

Cardiovascular diseases (CVDs) are the leading cause of death globally, with the prevalence of ischemic/coronary heart disease and stroke [1]. This group of diseases seems to typically affect middle-aged and elderly patients; however, younger and younger patients are being diagnosed with such conditions. Although the risk depends on many individual and environmental factors—dietary, socioeconomic, geographical, genetic, etc.—it is estimated that most cases of CVDs are, to some extent, preventable [2]. Keeping in mind the current threatening statistics of obesity, hypertension, and hypercholesterolemia among children and teenagers, it is urgent to emphasize the need for CVD prevention and its prospective benefits.

CVD diagnostics are mostly based on structural changes in the vessels or/and heart, as well as biochemical markers. New biomarkers are constantly being listed to make CVD control and diagnostics more effective and sensitive from the early stages and the first symptoms. Markers of endothelial dysfunction and low-grade inflammation, such as high-sensitivity C-reactive protein (hsCRP), have been included cardiovascular risk detection. hsCRP is a positive marker of unstable atherosclerotic plaques that is commonly used in the diagnosis and treatment of CVDs [3]. Adiponectin is a protein adipose tissue-derived hormone with anti-inflammatory and vasoprotective properties, opposite to hsCRP. A decreased adiponectin level is reported to correlate with a higher risk of CVD, metabolic syndrome, atherosclerosis, type 2 diabetes mellitus, and infarction [4,5,6].

Children with myelomeningocele (MMC) are a specific and very heterogenous group. This severe form of spina bifida is most often accompanied by hydrocephalus, impaired motor function due to paralysis of the lower limbs, neurogenic bladder (NB), and slow colonic transit [7]. It is important to highlight that individuals with MMC appear more prone to both congenital and acquired CVDs, which are also reported as one of the leading causes of death among adults with MMC [8]. Additionally, patients with MMC are at high risk of renal function deterioration and are likely to develop chronic kidney disease (CKD) in the future, which is significant given the well-established link between declining renal function and increased CVD risk. The literature on early CVD prevention in NB patients is sparse. Identifying early markers for predicting CVD risk in this population would represent a significant step forward in the prevention of CVDs. The aim of this study was to assess the risk of CVD in a group of children with MMC and a control group based on the serum levels of the antagonist-acting biomarkers adiponectin and hsCRP.

## 2. Materials and Methods

### 2.1. Patients

A prospective clinical estimation based on 87 children was conducted among the patients of the Department of Pediatrics and Nephrology of the Medical University of Białystok. The patients were divided into two subgroups: 67 NB children (33 girls and 34 boys) with a median age of 9.0 years and 20 healthy controls (8 girls and 12 boys) with a median age of 8.29 years. The exclusion criteria for both cohorts included the presence of active infection, diagnosed congenital cardiac anomalies, hypertension, and familial hypercholesterolemia. The control group comprised healthy children recruited during routine pediatric evaluations, with no documented history of renal or neurological disorders or CVD. These individuals adhered to a standard diet and were not receiving any pharmacological treatments or dietary additives.

Health status was determined based on the patients’ past medical history, parental reports, and routine laboratory tests to rule out the presence of acute inflammation. All of the data analyzed during the study were collected from the patients’ medical history and included the following: sex, age, somatometric parameters (height, weight, body mass index (BMI)). In the case of wheelchair-dependent children we used recumbent lengths as a standard equivalent of height measurement.

BMI was calculated using the WHO guidelines for sex and age. In both the NB children and the control group, lipid profile values (cholesterol, HDL, LDL, triglycerides) were assessed using laboratory standard blood sample tests.

Measurements of the plasma levels of hsCRP and adiponectin were performed in all the studied patients using commercially available monoclonal ELISA kits (Immundiagnostik AG, Bensheim, Germany) according to the manufacturer’s recommendations. Blood samples were collected to obtain serum after 12 h of fasting.

The levels of spinal lesion in patients with MMC were assessed clinically and, according to intraoperative findings or radiological findings from neurological examination, they were classified into three groups: thoracolumbar, lumbosacral, and sacral lesion.

Each patient’s walking ability and motor function were defined as the lowest level on the better side at which the child was able to perform an antigravity movement through the available range of joint motion, according to the 4-grade Hoffer scale (HS) presented in Table 1 [9].

### 2.2. Statistics

The data were collected in a Microsoft Excel database. Statistical analysis was performed using Statistica 13.3. (StatSoft Inc., Tulsa, OK, USA). Normal distribution of data was tested with the Shapiro–Wilk W test and then statistical analysis was performed using non-parametric tests (Mann–Whitney and Spearman). A *p*-value < 0.05 was considered statistically significant.

### 2.3. Ethical Issues

Written informed consent was obtained from all the enrolled subjects, subsequent to receiving full information about the study. The study was approved by the Ethics Committee of the Medical University of Bialystok and conducted in accordance with the Declaration of Helsinki.

## 3. Results

The clinical and biochemical characteristics of the study participants and comparisons between both groups are listed in Table 2.

Adiponectin and hsCRP levels were higher in the MMC patients and the differences were statistically significant (*p* = 0.001 and *p* = 0.035, respectively). In contrast, no significant differences were found between the groups in terms of age or somatometric parameters. Additionally, adiponectin and hsCRP levels did not differ between boys and girls (*p* = 0.97 and *p* = 0.29, respectively).

The study revealed that the NB patients classified into the wheelchair-dependent group (HS1) were the most heterogenous in terms of somatometric parameters. The number of NB patients diagnosed as underweight or obese was the same (17.91%), although nearly half (49.25%) of the NB patients remained in the norm of WHO percentile charts. There were no underweight children in the control group. We found statistically significant negative correlations between adiponectin levels and BMI, body mass, nutritional status, and systolic blood pressure, as well as positive correlations with total cholesterol, in the MMC patients (−0.564, −0.649, −0.453, −0.451, 0.403, respectively). The detailed somatometric assessment of the study groups according to WHO standards is presented in Table 3.

The differences in adiponectin and hsCRP levels according to nutritional status are included in Table 4.

In overweight MMC patients, adiponectin levels were the lowest, significantly lower than those in the control group. Conversely, underweight patients exhibited the highest adiponectin levels. In contrast, hsCRP levels followed an opposite trend, with the lowest values found in the underweight group and the highest observed in both the overweight and obese groups.

**Figure 1:**
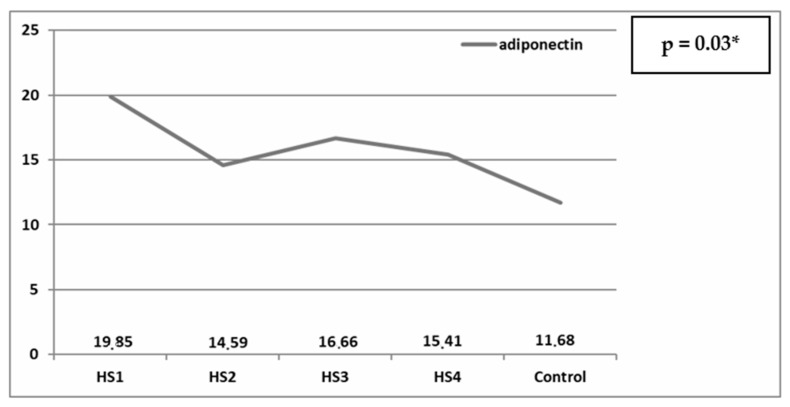
Median values of adiponectin (ug/mL) in HS groups and control group. * *p* < 0.05.

Figure 1 displays the median adiponectin levels in the MMC patients, categorized according to HS (Chi^2^ = 8.95, *p* = 0.03).

We applied the cardiovascular risk classification for hsCRP levels based on the ELISA test manufacturer’s guidelines: hsCRP values below 1.0 mg/L indicate low risk for CVDs, values between 1.0 and 3.0 mg/L represent moderate risk, and values above 3.0 mg/L suggest high risk. The distribution of hsCRP values among NB patients according to this classification is presented in Table 5.

**Figure 2:**
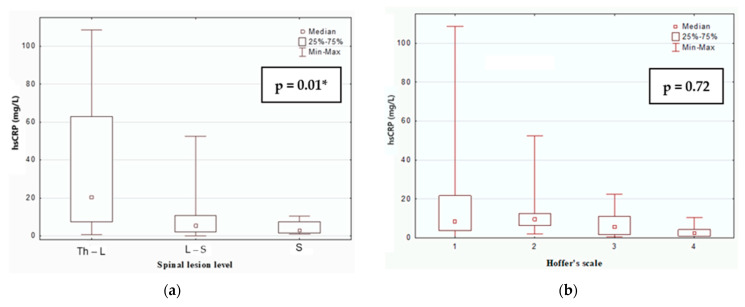
Median hsCRP levels in MMC patients, categorized by lesion level (**a**) and HS (**b**). * *p* < 0.05.

Figure 2 illustrates the median hsCRP levels in MMC patients, categorized by lesion level (Chi^2^ = 9.05, *p* = 0.01) and ambulatory function according to HS (Chi^2^ = 1.33, *p* = 0.72), respectively. The highest levels of hsCRP were detected in MMC patients with a Th lesion level and in non-walker patients. We found a statistically positive correlation between hsCRP and BMI (r = 0.279, *p* < 0.05).

## 4. Discussion

Despite the clinical heterogeneity among patients with MMC, they appear to be more susceptible to a range of disorders due to predisposing factors associated with both congenital and acquired CVDs [10]. The key risk factors contributing to cardiovascular complications in this population include motor impairments, which may lead to reduced blood circulation, weight gain, and dyslipidemia. Autonomic nervous system dysfunction can further impair heart rate regulation and blood pressure control [11]. Additionally, NB is a significant risk factor for CKD, and recurrent urinary tract infections may promote systemic inflammation, a recognized contributor to CVD risk. Although not directly caused by MMC, children with NB may present with coexisting congenital heart anomalies. In our study we evaluated established serum markers of CVDs in this population, which appears to be a valuable approach for the early identification and management of potential complications.

Our analysis demonstrated statistically significant differences between patients with MMC and the reference group concerning both evaluated biomarkers. Adiponectin, a protein synthesized predominantly by adipocytes, plays a crucial role in modulating various metabolic pathways, particularly those involving carbohydrates and lipids. In our investigation, adiponectin levels were elevated in the MMC cohort compared to the control group. Clinical evidence underscores the context-dependent and occasionally contradictory physiological and pathological roles of adiponectin [12]. In the majority of studies, circulating adiponectin concentrations exhibit an inverse relationship with obesity, type 2 diabetes mellitus, and CVDs. Beauloye et al. [13] proposed that adiponectin levels are associated with the initial stages of atherosclerotic plaque formation, suggesting their potential utility as a reliable and independent biomarker. Conversely, serum adiponectin concentrations are markedly elevated in conditions such as heart failure and CKD, where increased adiponectin levels have been linked to heightened risk of both all-cause and cardiovascular mortality [14,15]. This phenomenon, often referred to as the “adiponectin paradox,” remains insufficiently understood. Potential contributing factors for altered adiponectin levels in MMC patients include disrupted lipid metabolism, decreased mobility, enhanced adiponectin synthesis by adipose tissue, impaired renal function, and other unidentified physiological processes. In our study, nutritional status and ambulatory capacity emerged as relevant variables potentially influencing adiponectin concentrations. Paradoxically, adiponectin levels are inversely related to the amount of body fat—especially visceral fat [13]. Based on somatic assessments, the MMC cohort was notably heterogeneous. These children exhibited increased vulnerability to malnutrition due to gastrointestinal dysfunction, while simultaneously being predisposed to overweight and obesity as a result of impaired motor function. In our MMC patient cohort, the majority of children were classified as underweight, and these individuals exhibited higher adiponectin levels compared to those categorized as overweight or obese. This, combined with other disease-related factors, may contribute to elevated CVD risk, as malnutrition adversely impacts cardiovascular health across the lifespan and is associated with an increased likelihood of coronary artery disease or hypertension. In contrast, no participants in the control group were classified as underweight, which may partially explain the statistically significant differences in adiponectin levels observed between the groups. Furthermore, reduced mobility appears to be an additional factor associated with elevated adiponectin levels, as children reliant on wheelchairs exhibited the highest concentrations.

In addition, higher total cholesterol levels were observed in the study group compared to the controls. Although the difference approached statistical significance, it did not reach conventional levels. Additionally, a significant positive correlation was found between adiponectin and total cholesterol levels. The relationship between serum adiponectin and cholesterol is complex. In healthy individuals, higher adiponectin levels are consistently linked to higher HDL levels [16]. However, in certain diseases, like type 2 diabetes or metabolic syndrome, positive correlations between adiponectin and total cholesterol or LDL have been observed [17,18]. The relationship between adiponectin and total cholesterol seems to be more complex and may depend on the distribution of lipoprotein subclasses and other metabolic factors.

hsCRP is a proinflammatory cytokine used to assess unstable atherosclerotic plaques and the development of CVDs. It is more susceptible to environmental and general medical condition factors than adiponectin. Patients with CKD have been reported to present elevated levels of serum hsCRP in comparison to a healthy population [19,20]. A recent study by Goryia et al. [21] demonstrated a positive association between increasing serum creatinine levels and rising hsCRP concentrations in CKD patients. In our study we detected increased hsCRP levels in the study group compared to the controls, and this may suggest that this group is at risk of kidney function deterioration. MMC patients are exposed to multiple risk factors, including recurrent urinary tract infections and NB [22,23]. Therefore, early identification and prevention of CKD is crucial, aligning with the Latin adage “*Morbum evitare quam curare facilius est*”—it is easier to prevent disease than it is to treat it.

In our study, we identified statistically significant differences in hsCRP levels among children when stratified by motor function and the anatomical level of spinal lesion. Specifically, patients with thoracolumbar spinal injuries exhibited the highest hsCRP concentrations. Furthermore, similar trends were observed with respect to motor function, as wheelchair-dependent children showed the highest serum hsCRP levels. These findings align with established evidence suggesting that reduced mobility contributes to increased CVD risk. Notably, our cohort consisted of relatively young patients (median age: 9 years) without clinical signs of cardiovascular disease. Despite this, they already showed inflammatory markers suggestive of early cardiovascular risk.

Another very important aspect is bladder function in MMC patients. Numerous studies have reported elevated hsCRP levels in individuals with impaired bladder function, highlighting the role of chronic inflammation in this population [24,25,26,27]. Of particular relevance is the correlation demonstrated by Hsiao et al. [28] between increased serum CRP and reduced maximum urinary flow rate, suggesting that bladder dysfunction may be secondary to inflammation of the detrusor muscle or bladder outlet obstruction. Furthermore, reductions in CRP levels following anti-inflammatory treatment support the potential utility of CRP as a biomarker for both disease monitoring and treatment response in patients with lower urinary tract symptoms. In the longitudinal management of MMC patients with bladder dysfunction, regular assessment of inflammatory markers may be beneficial for the early identification and prevention of progressive bladder and renal impairment.

Notably, our analysis revealed significant correlations between the examined biomarkers and BMI, with a positive association observed for hsCRP and a negative association for adiponectin. These findings underscore the role of systemic inflammation linked to increased adiposity and its contribution to elevated CVD risk in overweight and obese pediatric populations. This finding emphasizes the need for effective weight management in these patients to mitigate inflammatory burden and the associated health risks.

We realized that our study had some limitations. First, the sample size was relatively small, particularly after stratification by HS and level of spinal lesion, which may have limited the statistical power of subgroup analyses. In relation to bladder function, combining hsCRP with additional biomarkers and urodynamic evaluations could yield a deeper understanding of inflammatory activity. In relation to renal function, we included serum creatinine and urea. Although creatinine is not an ideal indicator in children with MMC due to their reduced muscle mass, it remains a standard reference and offers some insight into renal status. Future research should aim to incorporate more reliable measures, such as cystatin C or eGFR. Furthermore, potential confounders such as medication use and diet were not comprehensively detailed, which may have influenced the inflammatory profiles observed. Given the multifactorial nature of cardiovascular risk, it is important to consider potential genetic contributions—particularly since MMC itself is a congenital condition. Finally, longitudinal follow-up, including repeated assessments over several years, would enhance the understanding of the progression and long-term implications of our findings.

## 5. Conclusions

The findings of our study suggest that children with MMC may exhibit early markers of cardiovascular risk, as indicated by elevated levels of hsCRP and altered adiponectin concentrations. We would like to highlight that:

1. Increased hsCRP levels may be considered risk factors of CVD in MMC children.
2. Altered adiponectin levels in MMC patents appear to be influenced by nutritional status, body composition, and mobility limitations.
3. Overweight patients with MMC have the lowest adiponectin levels and the highest hsCRP concentrations when compared to healthy controls.
4. The risk of CVD in MMC children may correlate with the level of spinal lesion, with higher lesion levels associated with increased cardiovascular risk.

## Figures and Tables

**Table 1:** Hoffer scale (HS) ambulation scoring [9].

Score	Name	Criteria
HS1	Non-walker	Wheelchair-dependent
HS2	Exercise walker	Walking only in therapeutic situations
HS3	Household walker	Using braces or crutches for indoors and using wheelchair outdoors
HS4	Community walker	Ambulating outdoors with or without braces but using wheelchair

**Table 2:** Characteristics and comparison of study groups. NB, neurogenic bladder; BMI, body mass index; HDL, high-density lipoprotein; LDL, low-density lipoprotein; hsCRP, high-sensitivity C-reactive protein.

Variable	NB Group	Control Group	*p* Value
	Median (minimum–maximum)		
Age (years)	9.0 (0.75–17.7)	8.29 (1.42–17.75)	0.94
Height (cm)	130 (7–165)	127 (78–179)	0.49
Body mass (kg)	28 (7.4–84.8)	26.5 (9.15–79)	0.71
BMI (kg/m^2^)	16.89 (10.33–31.5)	16.4 (13.5–29.4)	0.99
Systolic blood pressure (mmHg)	107.45 (85–141)	93.1 (73–120)	0.99
Diastolic blood pressure (mmHg)	67.6 (50–101)	57.9 (41–70)	0.52
Triglycerides (mg%)	87.7 (30–302)	90.6 (48–243)	0.98
Total cholesterol (mg%)	181 (112–229)	168.5 (106–203)	0.09
HDL (mg%)	55 (40–83)	51.5 (39–64)	0.89
LDL (mg%)	102 (67–160)	100.5 (26–151)	0.36
Creatinine (mg/dL)	0.39 (0.15–0.8)	0.45 (0.18–0.76)	0.9
Urea (mg/dL)	25.5 (11–45)	27 (22–40)	0.89
Adiponectin (ug/mL)	16.7 (0.25–30)	11.4 (5.96–18.09)	0.001 *
hsCRP (mg/L)	9.09 (0.55–334.47)	4.07 (0.36–18.21)	0.035 *

* *p* < 0.05.

**Table 3:** Somatometric assessment according to Hoffer scale (HS) and control group.

	Underweight (<3 pc)	Normal (3–85 pc)	Overweight (85–97 pc)	Obese (>97 pc)	Total Number
	Number of patients (% of group)				
HS1	9 (26.47%)	13 (38.24%)	5 (14.7%)	7 (20.59%)	34
HS2	1 (8.33%)	6 (50.00%)	2 (16.67%)	3 (25.00%)	12
HS3	1 (11.11%)	5 (55.56%)	1 (11.11%)	2 (22.22%)	9
HS4	1 (8.33%)	9 (75.00%)	2 (16.67%)	0 (0.00%)	12
Total number	12 (17.91%)	33 (49.25%)	10 (14.92%)	12 (17.91%)	67
Control group	0 (0.00%)	16 (80.00%)	3 (15.00%)	1 (5.00%)	20

**Table 4:** Adiponectin and hsCRP serum levels in MMC patients depending on nutritional status.

	Adiponectin	hsCRP
	Median (minimum–maximum)	
Underweight	23.94 (19.85–29.9)	5.09 (0.14–52.6)
Normal	16.56 (8.18–28.85)	7.41 (0.05–16.97)
Overweight	7.29 (1.3–108.7)	14.57 (6.58–17.16)
Obese	15.54 (0.25–22.58)	9.09 (1.3–64.83)

**Table 5:** hsCRP CVD risk classification in NB children.

CVD Risk	Low	Average	High
Number of patients (%)	6 (9%)	15 (22%)	46 (69%)

## Data Availability

The data presented in this study are available on request from the corresponding author. The data are not publicly available for ethical and privacy reasons.

## Abbreviations

The following abbreviations are used in this manuscript:

MMC	myelomeningocele
NB	neurogenic bladder
CVDs	cardiovascular diseases
hsCRP	high-sensitivity C-reactive protein
BMI	body mass index
HDL	high-density lipoprotein
LDL	low-density lipoprotein
WHO	World Health Organization
HS	Hoffer scale
CKD	chronic kidney disease
